# Genetic population structure of sympatric and allopatric populations of Baltic ciscoes (*Coregonus albula *complex, Teleostei, Coregonidae)

**DOI:** 10.1186/1471-2148-10-85

**Published:** 2010-03-29

**Authors:** Thomas Mehner, Kirsten Pohlmann, Che Elkin, Michael T Monaghan, Barbara Nitz, Jörg Freyhof

**Affiliations:** 1 Leibniz-Institute of Freshwater Ecology and Inland Fisheries, Müggelseedamm 310, 12587 Berlin, Germany; 2 ETH Zurich, Forest Ecology, Universitätstrasse 16, 8092 Zurich, Switzerland; 3 Zoological State Collection, Münchhausenstr 21, 81247 Munich, Germany; 4 Institute of Epidemiology, Helmholtz Center Munich, German Research Center for Environmental Health, Ingolstädter Landstr 1, 85764 Munich/Neuherberg, Germany

## Abstract

**Background:**

Teleost fishes of the Coregonidae are good model systems for studying postglacial evolution, adaptive radiation and ecological speciation. Of particular interest is whether the repeated occurrence of sympatric species pairs results from *in-situ *divergence from a single lineage or from multiple invasions of one or more different lineages. Here, we analysed the genetic structure of Baltic ciscoes (*Coregonus albula *complex), examining 271 individuals from 8 lakes in northern Germany using 1244 polymorphic AFLP loci. Six lakes had only one population of *C. albula *while the remaining two lakes had *C. albula *as well as a sympatric species (*C. lucinensis *or *C. fontanae*).

**Results:**

AFLP demonstrated a significant population structure (Bayesian *θ*^B ^= 0.22). Lower differentiation between allopatric (*θ*^B ^= 0.028) than sympatric (0.063-0.083) populations contradicts the hypothesis of a sympatric origin of taxa, and there was little evidence for stocking or ongoing hybridization. Genome scans found only three loci that appeared to be under selection in both sympatric population pairs, suggesting a low probability of similar mechanisms of ecological segregation. However, removal of all non-neutral loci decreased the genetic distance between sympatric pairs, suggesting recent adaptive divergence at a few loci. Sympatric pairs in the two lakes were genetically distinct from the six other *C. albula *populations, suggesting introgression from another lineage may have influenced these two lakes. This was supported by an analysis of isolation-by-distance, where the drift-gene flow equilibrium observed among allopatric populations was disrupted when the sympatric pairs were included.

**Conclusions:**

While the population genetic data alone can not unambiguously uncover the mode of speciation, our data indicate that multiple lineages may be responsible for the complex patterns typically observed in *Coregonus*. Relative differences within and among lakes raises the possibility that multiple lineages may be present in northern Germany, thus understanding the postglacial evolution and speciation in the *C. albula *complex requires a large-scale phylogenetic analysis of several potential founder lineages.

## Background

Fish in temperate lakes that have been formed since the last glaciation (12,000 - 15,000 yrs) provide good model systems for studying adaptive radiation and ecological speciation [1,2]. According to the ecological theory of adaptive radiation, environmental gradients and intense competition for scarce resources lead to divergent natural selection that, in turn, produces phenotypic differentiation [3]. Reproductive isolation is thought to evolve as a by-product of divergent natural selection because traits responsible for reproductive isolation are usually adaptive [4]. Sympatric populations of North temperate freshwater fish with significant genetic differentiation and strong ecological divergence are well known in sticklebacks (*Gasterosteus aculeatus *L.) [5], rainbow smelt (*Osmerus mordax *(Mitchill)) [6], Arctic char (*Salvelinus alpinus *(L.)) [7] or sockeye salmon (*Oncorhynchus nerka *(Walbaum)) [8]. But no group of fishes is as well known for adaptive radiations and complex speciation patterns in postglacial lakes as coregonid fishes (Teleostei: Coregonidae) [9].

A recent review [9] identified six potential modes of speciation in *Coregonus*: sympatric speciation from a single founder lineage, repeated invasions from a single lineage, hybrid swarm radiation, and three variants of allopatric speciation; complete or following secondary contact with either subsequent genomic reinforcement or with subsequent ecological reinforcement, depending on the levels of reproductive isolation between the taxa [9]. In order to distinguish from among these potential modes of speciation, population genetic information is required in addition to phylogenetic, biogeographical, morphological and ecological data. Specifically, lower genetic differentiation between sympatric than between allopatric populations provides evidence for hybrid swarm and sympatric speciation scenarios. Conversely, more pronounced reproductive isolation between sympatric populations than between allopatric populations provides evidence for allopatric speciation with reinforcement [9].

Ecological diversification leading to speciation has been repeatedly suggested in *Coregonus *[reviewed by [9]]; however, recent reviews [10,11] have challenged the assumption that all cases of ecological segregation between sympatric populations provide valid evidence for ecological speciation. Nevertheless, particularly strong support for sympatric ecological speciation can be obtained from cases of parallel ecological divergence and genetic differentiation in similar systems [12,13]. Further evidence for ecological speciation can be gained from genome scans that detect the same loci under selection in different systems with similar environments [14-16]. In a geographical context, the genetic distances among populations of the founder lineage of sympatric species should fit to an isolation-by-distance (IBD) model [17], whereas adaptive divergence between the sympatric populations may disrupt the IBD patterns [18]. Subsequently, sympatric populations would be genetically different but with a geographical distance of zero, thereby reducing our ability to detect significant IBD.

**Figure 1:**
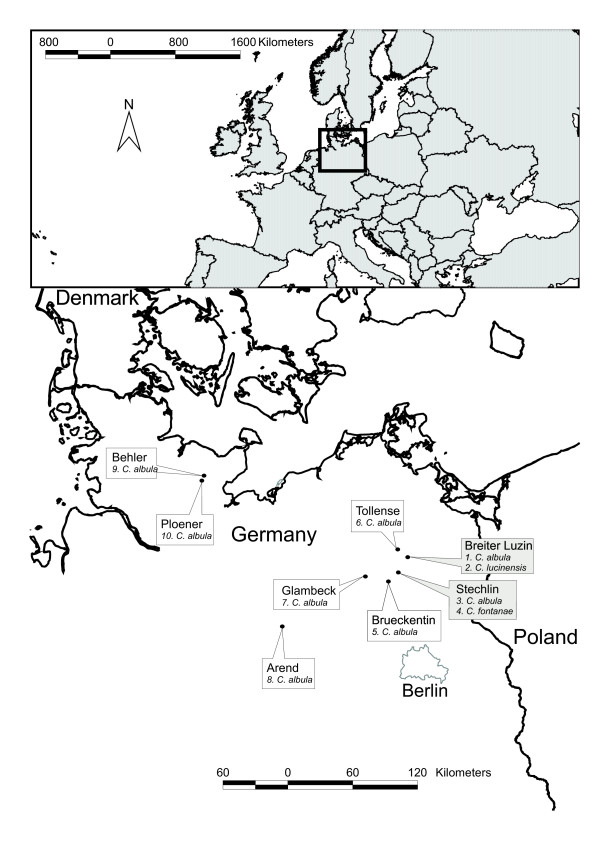
**Geographical map of sampling locations**. Geographical map showing the location of the 8 lakes in which the 10 populations were sampled (inset). Lakes with sympatric population are indicated in grey, and lakes with allopatric populations are indicated in white. The numbers refer to the population numbers in Table 1.

Within the Coregonidae, the postglacial evolution of whitefish (*Coregonus clupeaformis *(Mitchill) and *C. lavaretus *(L.) complexes) and the North America ciscoes (*C. artedi *Lesueur complex) are comparatively well studied [4,19-23]. Several speciation modes have been suggested for these groups, but evidence for parallel sympatric speciation from a single founder lineage is weak in any of the systems [9]. An alternative system to study putative parallel ecological speciation can be found within populations of the Baltic ciscoes (*C. albula *(L.) complex), a group that is widespread in northern Europe [24]. Sympatric populations of the *C. albula *complex occur in Scandinavia and Russia [25,26] and have been reported from two deep, stratified clear-water lakes [27] situated geographically close to one another in northern Germany (Fig. 1). In Lake Stechlin (Fig. 1), the autumn-spawning *C. albula *coexists with the smaller, spring-spawning *C. fontanae *[28]. Both species are pelagic zooplanktivores [29,30], but differ substantially with respect to temperature-dependent metabolic adaptations [31,32]. Analysis of six microsatellite loci revealed very low levels of genetic differentiation between the sympatric populations in Lake Stechlin [33]. In the nearby Lake Breiter Luzin (Fig. 1), two populations also coexist (*C. albula *and spring-spawning *C. lucinensis*) [34], between which the microsatellite differentiation was also very low [33]. While these results suggest a sympatric origin of each spring spawner, shared mtDNA haplotypes revealed lower genetic relatedness between sympatric taxa [33]. An important advance to our understanding of the origin of the sympatric pairs could come from a genetic comparison with other allopatric populations from the same geographical area. Furthermore, the few neutral microsatellite markers employed did not allow for the study of potentially parallel adaptive divergence in both lakes. Accordingly, the specific speciation mode of the sympatric populations has yet to be unambiguously determined.

Here, we studied genetic differentiation within the *C. albula *complex from eight lakes using amplified fragment length polymorphism (AFLP). Six lakes had single, autumn-spawning *C. albula *populations, whereas two lakes contained *C. albula *together with a sympatric species (spring-spawning *C. fontanae *in Lake Stechlin and *C. lucinensis *in Lake Breiter Luzin) (Fig. 1). We first examined the overall genetic structure of the 10 *Coregonus *populations, with particular emphasis on comparing differentiation between allopatric and sympatric populations. Under the scenario of sympatric speciation, we expected to find greater genetic similarity between the sympatric populations within lakes than among lakes [33]. Species that have evolved recently may not have developed full genetic incompatibilities and thus may hybridize with little intrinsic fitness loss, therefore we also tested whether ongoing hybridization between sympatric populations may blur historical differentiation [35]. Second, we fit genetic distances to a stepwise IBD model and hypothesized a disruption of IBD if sympatric populations are included [18]. Third, we calculated the probability of hybridization between sympatric populations and fish from putative stocking source populations. Enhancement stocking in the commercially important *Coregonus *lake populations is a common management practice [36-38], and our earlier work has quantified how this can modify the genetic identity of the target populations and disrupt IBD [39]. We hypothesized that hybridization with *C. albula *stocked from allopatric autumn-spawning populations would be more likely for the autumn-spawning *C. albula *from both lakes than for the sympatric spring-spawning *C. fontanae *or *C. lucinensis*. Finally, we tested for the presence of outlier loci that were shared in both sympatric pairs to find genetic evidence for parallel adaptive divergence [15,16].

## Results

### All Coregonus populations and all AFLP loci

Using a multiplex AFLP method (see Methods) we scored 1264 loci from 271 individuals, of which 1244 (98.4%) were polymorphic over the 10 populations. Differentiation (*θ*) between the repeated samplings of populations in Lake Stechlin were not significantly different from zero (*C. albula*: *θ *= -0.004, *p *= 0.999; *C. fontanae*, *θ *= -0.007, *p *= 0.99; 50175 iterations in ARLEQUIN), thus samples were pooled for each population in subsequent analyses. The percentage of polymorphic loci varied from 64 - 92% within the 10 populations (Table 1). Estimated with *h*_s_, the expected heterozygosity ranged from 0.219 to 0.300, with the highest values being found in the two *C. albula *populations that occur with the sympatric species (Table 1).

**Table 1:** The ten sampled populations of the *Coregonus albula *complex

No.	Lake/Population name	Geographical position		*n*	% polymorphic Loci	h_s_	95% credibility interval of h_s_	
		**North**	**East**				**lower**	**upper**
1	Lake Breiter Luzin - *C. albula*	53°21'	13°28'	23	91.5	0.300	0.296	0.304
2	Lake Breiter Luzin - *C. lucinensis*	53°21'	13°28'	27	82.4	0.232	0.229	0.236
3	Lake Stechlin - *C. albula*	53°09'	13°01'	38	85.9	0.278	0.275	0.282
4	Lake Stechlin - *C. fontanae*	53°09'	13°01'	32	84.9	0.228	0.225	0.231
5	Lake Brueckentin	53°15'	13°14'	17	63.6	0.224	0.219	0.228
6	Lake Tollense	53°30'	13°12'	32	74.4	0.258	0.254	0.262
7	Lake Glambeck	53°15'	12°37'	30	70.3	0.219	0.216	0.222
8	Lake Arend	52°53'	11°28'	30	91.4	0.249	0.245	0.252
9	Lake Behler	54°10'	10°28'	12	90.9	0.244	0.240	0.249
10	Lake Ploener	54°05'	10°24'	30	80.2	0.236	0.233	0.239
				Σ = 271	Total = 98.4	H_s _= 0.247	0.246	0.248

Lake names, geographical position and genetic diversity of the 10 *Coregonus albula *complex populations sampled. Allopatric populations consist exclusively of *C. albula*. Sympatric populations are named by lake origin and the lake-specific species names. *n *= number of individuals analysed, % polymorphic loci refers to the total of 1264 loci analysed, h_s _= gene diversity with credibility intervals calculated by a Bayesian approach as implemented in HICKORY, H_s _= the mean within-population expected heterozygosity (= Nei's gene diversity within populations).

There was significant genetic differentiation among the 10 populations, with 22.52% of variation found among populations (77.48% within populations; AMOVA, d.f. = 9.261, *p *< 0.0001). Global values of *θ *(0.225, *p *< 0.0001) and *θ*^B ^(0.222; credibility interval 0.216 - 0.228 95%) were nearly identical and also showed significant population structure. All pairwise *θ*-values were significantly different from zero (*p *< 0.0001) and ranged from 0.041 (*C. albula *from Lakes Stechlin and Breiter Luzin) to 0.405 (*C. albula *from Lakes Brueckentin and Behler) (see additional file 1: AdditionalFile_FST_Matrix).

**Figure 2:**
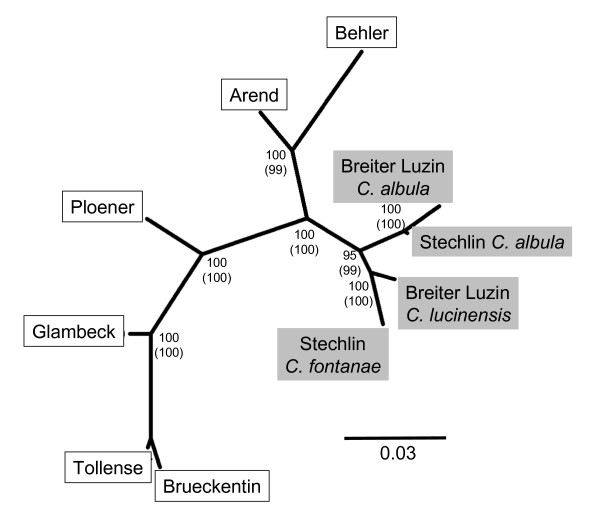
**Tree of genetic distances between *Coregonus *populations**. Unrooted neighbor-joining tree of Nei's genetic distances between 10 populations of the *Coregonus albula *complex. Allopatric populations consist exclusively of *C. albula *and are named according to the lake of origin (indicated by white colour). Sympatric populations are named by lake origin and the lake-specific species names (indicated by grey colour). Bootstrap support (%) from 100,000 iterations is provided for each node. Values in parentheses report support after removal of outlier loci.

All nodes of the NJ tree of Nei's genetic distances were supported by bootstrap values of at least 95% (Fig. 2). The tree grouped four lakes with single populations (Brueckentin, Glambeck, Tollense, Ploener) as distinct from the two lakes with sympatric population pairs (Stechlin, Breiter Luzin) (Fig. 2). Lakes Arend and Behler appeared more closely related to Stechlin and Breiter Luzin. Interestingly, the allopatric spring-spawners, *C. fontanae *and *C. lucinensis*, grouped more closely to one another than either did to its sympatric *C. albula *population (Fig. 2).

Pairwise genetic and geographical distances were not correlated for the entire dataset (Mantel *r *= 0.26, *n *= 45, *p *= 0.056), providing evidence that an IBD model did not appropriately describe the pattern of genetic differentiation among all 10 populations (see additional file 2: AdditionalFile_IBD_Models). An IBD model was also not appropriate to describe the genetic differentiation between all eight *C. albula *populations including those from Lakes Stechlin and Breiter Luzin (Mantel *r *= 0.31, *n *= 28, *p *= 0.086). In contrast, when only the six lakes with only a *C. albula *population were included, genetic distances increased significantly with geographical distance as predicted by an IBD model (Mantel *r *= 0.53, *n *= 15, *p *= 0.042). However, the pairwise difference between the closely located Lakes Ploener and Behler (Fig. 1) appeared to be slightly higher (0.23) than predicted by strict linear pattern (see additional file 2: AdditionalFile_IBD_Models).

### Sympatric pairs

Pairwise differentiation (*θ*^B^) within and among the sympatric population pairs in Lakes Stechlin and Breiter Luzin ranged from 0.028 - 0.113 (Table 2). *C. fontanae *appeared most distinct, while *C. lucinensis *was equally differentiated from the three other populations (Table 2). The lowest differentiation observed was between allopatric *C. albula *in Stechlin and Breiter Luzin (Table 2). This was significantly lower (i.e., outside the 95% credibility interval) than the differentiation with either sympatric taxon (*C. fontanae *in Lake Stechlin; *C. lucinensis *in Lake Breiter Luzin.

**Table 2:** Pairwise genetic differentiation for sympatric populations of Lakes Stechlin and Breiter Luzin.

	Lake Breiter Luzin *C. albula*	Lake Breiter Luzin *C. lucinensis*	Lake Stechlin *C. albula*	Lake Stechlin *C. fontanae*
Lake Breiter Luzin *C. albula*		0.063 (0.055-0.071)	0.028 (0.023-0.033)	0.113 (0.102-0.126)
Lake Breiter Luzin *C. lucinensis*	0.049 (0.042-0.057)		0.063 (0.055-0.072)	0.054 (0.047-0.062)
Lake Stechlin *C. albula*	0.027 (0.022-0.033)	0.052 (0.045-0.060)		0.083 (0.074-0.092)
Lake Stechlin *C. fontanae*	0.091 (0.081-0.103)	0.043 (0.036-0.050)	0.043 (0.037-0.052)	

Genetic differentiation (*θ*^B ^with 95% credibility intervals in parentheses) for the two sympatric population pairs of the *Coregonus albula *complex. Values were calculated with HICKORY based on all 1264 AFLP loci (above diagonals) and after removal of *n *= 95 loci putatively under selection at *p *= 0.95 (below diagonals).

**Figure 3:**
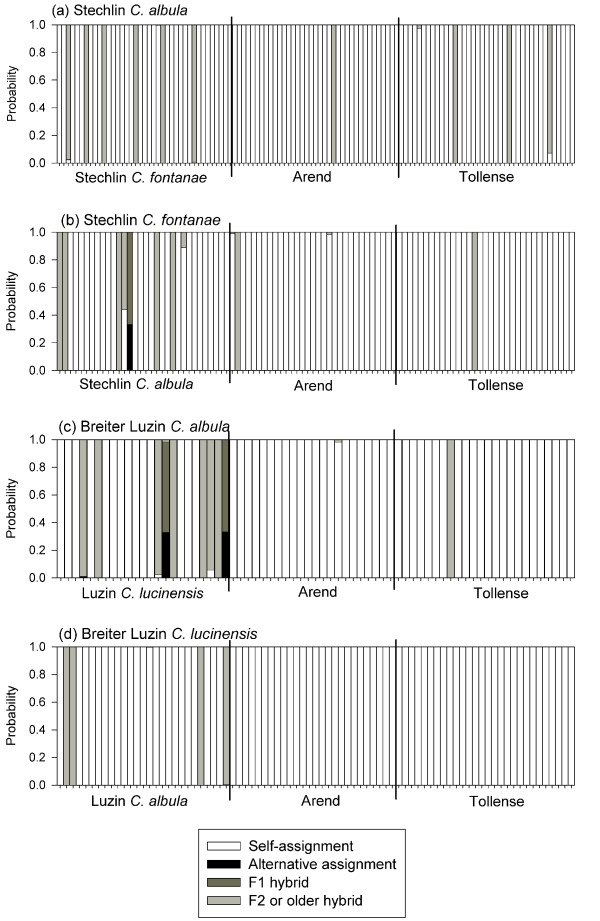
**Admixture analysis of hybridization for Lakes Stechlin and Breiter Luzin**. Results of the admixture analysis with limited prior probability of hybridization by STRUCTURE for the two sympatric population pairs, Stechlin *Coregonus albula *(a), Stechlin *C. fontanae *(b), Breiter Luzin *C. albula *(c) and Breiter Luzin *C. lucinensis *(d). Probability of admixture was estimated for the sympatric populations in each lake, and for Lakes Arend and Tollense as potential stocking sources. Hybrids refer either to the first (F1) or the second and older (F2) parental generations.

The analyses of potential gene flow within the two lakes by STRUCTURE found some evidence for the existence of hybrids between sympatric populations (Fig. 3). The mean proportion (*P*) of any individual's genome that originated from its own population [40] was generally ≥ 87% (Lake Stechlin: *C. albula*, *n *= 38, *P *= 96%, *C. fontanae*, *n *= 32, *P *= 94%, Lake Breiter Luzin: *C. albula*, *n *= 23, *P *= 87%, *C. lucinensis*, *n *= 27, *P *= 96%). Three individuals (one *C. fontanae *and two *C. albula *from Breiter Luzin) showed elevated probabilities of misclassification (alternative assignment) or hybridization in the F1-generation (Fig. 3). For 23 individuals, there was a high probability of F2- or older hybridizations with the sympatric population. In contrast, the probability of F1-hybrids formed with stocked fish from Lakes Arend and Tollense was zero for all populations, and only six individuals showed a high probability of F2- and older hybrids with stocked fish (Fig. 3).

### Outlier loci

**Figure 4:**
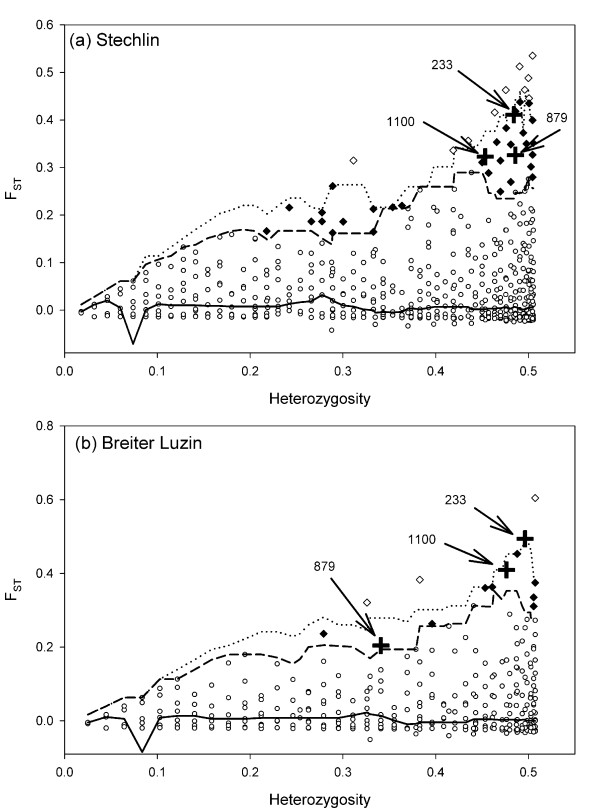
**Scatter plots of AFLP loci putatively under selection**. Explorative genome scans comparing differentiation of AFLP loci to the expected neutral distribution under *p *> 0.99 and *p *> 0.95 for Lake Stechlin (a) and Lake Breiter Luzin (b). Loci positively exceeding expected neutral F_ST_s are indicated by white diamonds (*p *> 0.995) or black diamonds (*p *> 0.975). Loci shared from both lakes are numbered and indicated by black crosses. Lines indicate the median (solid), the 97.5% quantile (dashed) and the 99.5% quantile (dotted). Some symbols represent multiple loci with almost identical coordinates.

The explorative genome scans revealed very few loci to be positively exceeding neutral expectations (at *p *= 0.995) in the two lakes with sympatric population pairs. More loci under putative selection were found in Lake Stechlin (*n *= 12, *ca*. 0.95%; Fig. 4a) than in Lake Breiter Luzin (*n *= 3, *ca*. 0.24%; Fig. 4b). No single locus was non-neutral in both lakes. At the *p *= 0.975 quantile, 5.30% of loci (*n *= 67) were positively non-neutral in Lake Stechlin, and 2.21% (*n *= 28) in Breiter Luzin. At this quantile, three loci (numbers 233, 879, 1100) were shared from both lakes (Fig. 4a, b).

After removal of all 95 loci putatively under selection above the *p *= 0.975 quantile in either Lake Stechlin or Breiter Luzin, genetic differentiation based on the remaining loci declined considerably, in particular between the sympatric pairs (Table 2). *θ*^B^-values were almost identical between *C. albula *and *C. fontanae *in Lake Stechlin (0.043) and *C. albula *and *C. lucinensis *in Lake Breiter Luzin (0.049), and did not differ from the differentiation between *C. fontanae *and *C. lucinensis *(0.043) (Table 2). Only differentiation between both *C. albula *populations was still lower (0.027), with its credibility interval not overlapping with those of any other pairwise comparisons (Table 2).

Genetic distances between the eight *C. albula *populations remained almost unaffected by the removal of outlier loci. The NJ-tree calculated from the putatively neutral AFLP loci had an identical topology to the NJ-tree based on all AFLP loci (Fig. 2), with only minimal changes in branch lengths (results not shown).

## Discussion

### Conflicting evidence for sympatric speciation

Our results suggest that the post-glacial evolution of sympatric *Coregonus *pairs studied here is more complex than previously thought. Neither spring spawner occurred elsewhere in allopatry, supporting the hypothesis that they evolved by sympatric speciation from the autumn-spawning *C. albula *within each lake, as suggested previously [33]. Nonetheless, the pattern of genetic differentiation that we observed contradicts this hypothesis. In cases of sympatric speciation, sympatric taxa should be more closely related [9], but here the autumn-spawning *C. albula *were more similar to each other than either was to its sympatric spring spawner. This was evident from the tests of pairwise population differentiation as well as from the NJ-tree based on genetic distance. These results were similar to an allozyme study of 19 Finnish lakes, where allopatric autumn spawners formed a separate group to winter- or spring spawners [41].

The complexity of the evolutionary history is evidenced by the somewhat conflicting results of previous genetic analyses. Six microsatellite loci revealed greater genetic similarity between sympatric spring- and autumn-spawners within lakes, thus suggesting ongoing gene flow and a sympatric origin [33]. In contrast, mtDNA from the same study [33] qualitatively support the AFLP results here, that differentiation between sympatric taxa is greater than between allopatric taxa. In particular, the two most abundant of four recorded haplotypes were shared by *C. albula *in both lakes. One of these was shared with the Stechlin sympatric *C. fontanae *and the other was shared with the Lake Breiter Luzin sympatric *C. lucinensis*. *C. fontanae *and *C. lucinensis *shared no haplotypes [33].

The reason for the discrepancy between the microsatellite and AFLP data in one of the population pairs is not clear. The large number of AFLP loci employed here (>1200) should provide a better estimate of divergence than microsatellite loci (6), although we found evidence that not all loci were strictly neutral. The two marker systems produced similar estimates of genetic structure for populations of brown trout (*Salmo trutta *L.) in 11 Norwegian lakes, although admixture was greater based on AFLP [42]. AFLP results were more congruent than mtDNA with morphological characters in mormyrid electric fishes, likely due to mitochondrial introgression among lineages [43]. Accordingly, AFLP seem to be an appropriate tool to examine the genetic differentiation of incipient ecological speciation [44]. But as noted above, our results suggest that not all AFLP markers were strictly neutral. Removing the loci putatively under selection reduced the within-lake genetic distances considerably, and differentiation based on the remaining neutral AFLP loci was more similar to the pattern obtained from the neutral microsatellite data. Interestingly, the genetic distance to other, allopatric *C. albula *populations remained largely unaffected. Previous studies similarly found that differentiation between ecologically or morphologically distinct populations can be maintained for a small portion of the genome, whereas gene exchange continues to prevent divergence at the majority of loci [16,45].

Only three outlier loci occurred in both sympatric pairs at *p *> 0.975. It is possible that such a small number of loci positively exceeding neutral expectations might have occurred just by chance, thus providing little evidence in support of the hypothesis that the sympatric pairs result from parallel sympatric speciation [12]. Alternatively, it may be that only a few genes are involved. The few shared loci deserve further attention since recent analyses in the *C. clupeaformis *complex in North America indicated that these *θ *outliers can be significantly clustered around quantitative trait loci (QTL) involved in ecologically based reproductive isolation [46-48]. Proportions of outlier loci substantially exceeding 1% were found in other studies applying a similar pairwise approach [13,15,16,49,50].

The presumed modes of ecological segregation have been well studied in Lake Stechlin, where sympatric taxa (*C. albula *and *C. fontanae*) perform diel vertical migrations with different amplitudes [51,52] and they differ in their final temperature preferendum and their metabolic costs of swimming [31,32]. The commonly observed benthic-limnetic habitat and diet segregations typical for whitefish do not occur in Stechlin [29]. Such a pattern of physiological segregation but low trophic differentiation presumably exists for the species pair in Lake Breiter Luzin, since both lakes are deep, with steep temperature gradients and overall low productivity [27]. Recent data suggest *C. albula *and *C. lucinensis *in Breiter Luzin have similar diets [53], as in Lake Stechlin [29]. However, adaptive divergence may be weaker in Lake Breiter Luzin than in Lake Stechlin. For example, *C. lucinensis *and *C. albula *do not show the same strong vertical microhabitat segregation (S. Schiller, Leibniz-Institute of Freshwater Ecology and Inland Fisheries, unpubl. results) as found between *C. fontanae *and *C. albula *in Lake Stechlin [51]. A weaker adaptive divergence is supported by the lower genetic distance and a substantially lower number of loci putatively under selection in Lake Breiter Luzin than in Lake Stechlin. Accordingly, these species pairs would resemble 'isolation by adaptation' (IBA), a process in which levels of genetic differentiation among population pairs will be positively correlated with degree of adaptive phenotypic divergence [13,16].

### Stocking and hybridization

An important part of our analysis was to determine whether the genetic similarity of Stechlin and Breiter Luzin *C. albula *was a result of modification by stocking and hybridization. Enhancement stocking of vendace populations by freshly hatched larvae (up to 6000 ind. ha^-1 ^annually) obtained primarily from two source populations (Lakes Arend and Tollense) has been practised in Germany for at least 50 years, with the aim of stabilizing natural recruitment and fisheries yield of sympatric and allopatric *C. albula *populations [39]. Because both putative stocking sources contain only autumn-spawning *C. albula *populations, hybridization with *C. albula *in Stechlin and Breiter Luzin was hypothesized to be more likely than hybridization with the spring-spawning *C. fontanae *and *C. lucinensis*. Our results support this hypothesis. Furthermore, our recent analysis of the genetic effects of stocking on 23 allopatric *C. albula *populations revealed a reduction of genetic distance by about 10% between lakes stocked intensively with a genetically similar source [39]. Accordingly, a weak secondary homogenization between Lakes Stechlin and Breiter Luzin by stocking from Lakes Arend or Tollense cannot be ruled out, but the evidence is not strong enough to conclude that the similarity of the autumn spawners is an exclusive effect of current fisheries management practices.

Hybridization within the lakes could also contribute to the observed patterns, whereby introgression of *C. albula *genetic polymorphisms may obscure any historical similarities of the two spring spawners. Because species that have evolved since the most recent glaciation about 12,000 years ago may not have developed full genetic incompatibilities, they may hybridize with little fitness loss [35]. The probability that single individuals have parents or grandparents from the other population was low, but greater than zero in all four populations. Therefore, gene flow between the sympatric populations may not be completely interrupted, despite the differing spawning times. Weak ongoing hybridization between the sympatric populations can also be inferred from the higher heterozygosity of sympatric compared with allopatric *C. albula *populations [54]. In this analysis, we used a high prior probability of pure ancestry similarly applied to the analysis of sympatric whitefish populations from the Canadian Cliff Lake [55]. This sympatric whitefish population pair was identified as being reproductively isolated by AFLP-markers (*θ*^B ^= 0.22) [4], but nevertheless encompassed seven out of 47 individuals with strong evidence for mixed ancestry [55]. Accordingly, occasional hybridization within the two sympatric populations of the *C. albula *complex studied here cannot be excluded.

### Large-scale genetic structure of Coregonus

An important finding was that the sympatric populations of Lakes Stechlin and Breiter Luzin were genetically distinct from the six other *C. albula *populations (Brueckentin, Glambeck, Ploener, Tollense, Ahrend, Behler). These patterns were confirmed by the IBD analyses, which demonstrated that increasing genetic distances corresponded to increasing geographical distances only for the six lakes with single *C. albula *populations. The interruption of the IBD became obvious when all eight *C. albula *populations were included, suggesting that the *C. albula *in sympatry with spring-spawners are genetically distinct from other *C. albula *populations. Even within the six allopatric *C. albula *populations, the IBD model was slightly biased by a higher-than-expected genetic distance between the neighboring Lakes Ploener and Behler. This is attributable to a higher genetic similarity of the population from Lake Behler to the sympatric populations and to *C. albula *from Lake Arend. The *C. albula *population in Lake Arend is not native, but has been introduced from Lake Enzig (Outer Pomerania, now Poland) about 100 years ago [56]. Historical introduction of *C. albula *in Lake Behler cannot be excluded, whereas recent stocking can be ruled out [39]. Altogether, the results from our analyses suggest a complex evolutionary scenario for the sympatric populations, including putative parallel sympatric speciation, introgression on secondary contact, divergent selection and within-lake hybridization.

Taken together, our results suggest the following evolutionary and phylogeographical hypothesis: All lakes in northern Germany including Lakes Stechlin and Breiter Luzin were invaded from a single refuge after the last glaciation about 12,000 years ago. This founder lineage of *C. albula *still exists in the majority of the lakes. The genetic differentiation between these populations can be appropriately described by an IBD model [this study] which is only slightly modified by more recent stocking [39]. In two of the deepest lakes (Stechlin and Breiter Luzin), parallel sympatric speciation along steep vertical abiotic gradients may have occurred. The putative sympatric speciation is suggested by the low within-lake genetic segregation of sympatric pairs by neutral markers [33]. Subsequent divergent selection may have induced a stronger genetic segregation of sympatric pairs, as suggested by the AFLP markers used here. Evidence for strong recent selection comes from the fact that the removal of AFLP loci putatively under selection substantially reduced the genetic differentiation of the sympatric pairs. Furthermore, physiological studies suggest that ecological divergence is driven by temperature-based adaptations of the sympatric pair at least in Lake Stechlin [31,32]. Recent stocking from identical autumn-spawning sources [39] may have slightly reduced the genetic distance between the *C. albula *populations in Lakes Stechlin and Breiter Luzin, as indicated by a low frequency of hybridization between these populations and *C. albula *from Lake Tollense. Secondary contact with another *Coregonus *lineage and subsequent introgression may have further shaped the genetic signature of the sympatric populations. Introgression is more likely for the autumn-spawning *C. albula *because the majority of all other European populations of the *C. albula *complex are autumn-spawners [24]. Ongoing within-lake hybridization between sympatric populations, as demonstrated here by the STRUCTURE analyses, may have resulted in the spreading of introgressed genetic material into both populations. Introgression after secondary contact may explain why both *C. albula *and the spring-spawning *C. fontanae *and *C. lucinensis *of Lakes Stechlin and Breiter Luzin were genetically distinct to the allopatric *C. albula *in the other German lakes.

The source of the secondary contact could be from populations living farther east. This is suggested by the similarity between Lakes Stechlin and Breiter Luzin and the population from Lake Arend, which has a historical origin in Poland. Brzuzan *et al. *[57] demonstrated that two distinct lineages exist within Polish *C. albula *populations, with one group occurring in the east, and one in central and west Poland. These authors speculated that these may have evolved by colonization from different refugia or by introgression from *C. sardinella *[57]. Interestingly, introgression from *C. sardinella *has also been suspected for the Lake Breiter Luzin populations [33]. An allozyme study [25] found low genetic distances between *C. sardinella *and *C. albula *populations in Russia. Accordingly, we cannot exclude that populations exist in East Europe which are part of a separate lineage involved into the secondary contact with the German sympatric populations, thus explaining the genetic similarity between Lakes Arend, Stechlin and Breiter Luzin. We would like to emphasize that this complex scenario is hypothetical at the moment. We cannot exclude that the sympatric populations had an allopatric divergence or originate from two different refugia [33], followed by reinforcement on secondary contact in Lakes Stechlin and Breiter Luzin. In this case, adaptive differentiation might have followed nonecological speciation [11]. Based on all available evidence from this and other studies, further clarification of speciation mode will require samples of the *C. albula *complex from several populations in Poland and Russia to be included in the analysis, as well as additional sympatric populations existing in Russia and Scandinavia [24]. Comparable evidence for multiple invasions as the main mechanism for adaptive divergence within single lakes was found for *C. clupeaformis *[58] and sticklebacks [59] in North America. Therefore, we cannot exclude that Russian or Scandinavian sympatric populations of the *C. albula *complex [25,26] underwent an allopatric stage as well.

## Conclusions

The effects of hybridization and stocking on the commercially important *Coregonus *can be quantified and accounted for, thus the group remains an excellent model for the study of speciation in sympatry. Despite having used more than 1200 marker loci in a study of 10 populations, our data failed to provide conclusive evidence for whether spring-spawning *Coregonus *stem from parallel sympatric speciation. Parallel trends in niche differentiation and phenotypic differentiation are evident in both lakes with sympatric populations, but neutral genetic differentiation suggests allopatric speciation and introgression on secondary contact. It is clear that population genetic criteria alone cannot resolve the mechanisms of speciation, and that our analysis benefited from a large spatial scale of sampling. Examination of relative differences within and among lakes has raised the possibility that multiple lineages may be present in northern Germany. As a result, phylogenetic analysis and sampling of additional lakes from potential source populations from a broad geographic range is required for an unequivocal identification of the evolutionary mechanisms and phylogeography which have led to the ecological and genetic diversity found in the *C. albula *complex.

## Methods

### Sampling

Ten populations of Baltic ciscoes were sampled from eight lakes in Germany from 2000 to 2004 (Fig. 1). In two of the lakes, *C. albula *occurs sympatrically with a spring-spawner; *C. fontanae *in Lake Stechlin and *C. lucinensis *in Lake Breiter Luzin. The remaining six populations of *C. albula *occur in allopatry in northern Germany, at variable geographic distances to Lakes Stechlin and Breiter Luzin (Fig. 1, Table 1). Two lakes (Arend, Tollense) were documented source populations for enhancement stocking for a large number of lakes in northern Germany [39]. The remaining four lakes (Glambeck, Brueckentin, Behler, Ploener) exhibit little or no genetic evidence of stocking [39], and are therefore considered as near-native populations. These lakes were included to determine whether the spring-spawning sympatric taxa (*C. fontanae*, *C*. *lucinensis*) occur elsewhere in allopatry.

In total, 271 individuals were analysed, with the number of analysed specimens per population varying between 12 and 38. To verify that all procedures and analyses produced consistent results, the Lake Stechlin populations were sampled twice, in 2000/2001 and 2007. These samples were later combined because the pairwise *θ *between the repeated samplings were not significantly different from zero (see results). All fish were caught with gill nets or trawls and stored on ice immediately after removal from the nets. Subsequently, clips from the pectoral fin were stored in pure ethanol.

### Genetic (AFLP) analysis

We examined population genetic diversity using amplified fragment length polymorphism (AFLP) in order to generate the large number of characters needed for genomic scans of non-model organisms [4,60]. We developed a multiplex method of fragment generation in order to increase the number of scorable bands in a given reaction. Total genomic DNA was extracted from pectoral fin tissue using the DNeasy Tissue Kit (Qiagen) according to the manufacturer's protocol. DNA-quality was visually inspected under UV-light on a 1% agarose-gel stained with ethidium bromide. For subsequent analysis, only samples with a clearly visible high molecular weight band were used. AFLP analysis was carried out following a slightly modified protocol of the AFLP-method [61]. Restriction and ligation were carried out in a single step in a thermocycler (2 h at 37°C, 8 h at 16°C). Genomic DNA (300-400 ng DNA per sample) was digested with restriction enzymes MseI (1 unit) and EcoRI (5 units). Polymerase chain reaction (PCR) adaptors specific to the cutting sites were ligated using DNA-Ligase (1 unit; all enzymes from New England Biolabs). Preselective PCR was performed with one selective base on each primer (MseI+C and EcoRI+A; all oligonucleotides from Metabion). PCR product quality was visually confirmed on an agarose gel after preselective amplification. For the selective amplifications, two additional bases were added to the 3' end of each primer (MseI/EcoRI^DYE^) und the utilization of two different fluorescent dyes permitted multiplexing, where two EcoRI primers were combined with one MseI primer (Table 3).

**Table 3:** Primers for the selective amplification step of AFLP

Primer combination	MseI Primer	EcoRI A	EcoRI B	Number of loci per primer pair
1	CAT	ACTfam	AAGjoe	208
2	CAG	ACTfam	AAGjoe	209
3	CTA	ACTfam	ACAjoe	214

All primers had 3 additional bases on the 3' end. EcoRI primers A and B were labelled with different fluorescent dyes (*fam *and *joe*) and 3 different multiplex reactions were run with two forward and one backward primer each.

Fragments were separated on an ABI Avant 3100 capillary sequencer with an internal size standard (GeneScan-500 LIZ; Applied Biosystems). Signal processing and binning were carried out using GeneMapper^® ^v4.0 (Applied Biosystems). Optimization yielded the following settings: bin width 1 bp, maximal peak width of 1.5 bp, scoring to presence (1) or absence (0) of fragments between 100 and 400 bp. Normalization to the sum of signal versus a fixed cut off value did not cause any differences, thus we used peak detection levels of 50 relative fluorescent units. Correct fit of the size standard was checked manually for all electropherograms. Larger fragments were excluded from the analyses to avoid errors from signal-strength drop off resulting from PCR or capillary loading bias. Only electrograms were evaluated which met the following criteria: smallest detected fragments < 50 bp and largest > 450, at least 5 fragments > 400 bp, no visible drop outs (signal remaining flat at base line for > 20 bp) in the entire spectrum, a minimum of 100 fragments in each primer combination and reproducible standards. We used 2 samples as standards in each PCR and subsequent analyses. They were considered reproducible only if they yielded > 95% fragment reproducibility to previous runs. Analyses of individual samples were repeated from restriction/ligation on until meeting the above described criteria. A second complete AFLP analysis of a random 10% of the samples yielded 96.08% reproducibility.

### Genetic diversity and structure of all Coregonus populations

Preparation of input data files for analyses was conducted by AFLPDAT[62]. Data on population genetic structure were summarized using the frequency of polymorphic fragments as calculated in AFLP-SURV[63]. The expected heterozygosity *h*_s_, the mean within-population expected heterozygosity *H*_s_, and their 95% credibility values, were calculated using a Bayesian approach with 250,000 generations after a burn-in of 50,000 generations implemented in HICKORY 1.1 [64]. To account for potential slight deviations from Hardy-Weinberg equilibrium, we ran the full model which calculates an inbreeding coefficient (F_IS_). Because this value can be unreliable [65], we report only the results on overall population differentiation here.

The genetic structure within and among all 10 populations was inferred from an analysis of molecular variance (AMOVA) and a global estimate of among-population differentiation (*θ*) [66], both calculated using ARLEQUIN 3.2 [67]. Pairwise *θ *was calculated for all population pairs and tested for significant deviation from zero by a numerical resampling procedure in ARLEQUIN. All analyses used 50,175 permutations. An overall estimate of genetic differentiation using a Bayesian approach (*θ*^B^) [68] was also calculated using HICKORY as described above (*θ*^II ^in the notation of the HICKORY manual). A modified Nei's genetic distance [69] was calculated between all population pairs in order to create an unrooted neighbor-joining (NJ) tree using PHYLIP 3.6 [70]. Subsequently, the distance matrix was bootstrapped 100,000 times using AFLP-SURV to calculate a majority-rule consensus tree in PHYLIP 3.6 [70].

An isolation-by-distance model was calculated by correlating the matrices of log_10 _direct geographical distances (km) and pairwise genetic distance (*θ*/1-*θ*) [66] between the lakes by a Mantel test (*n *= 45 pairwise comparisons, PC-ORD 5.01 [71]). The geographical distances between the sympatric populations in either Lakes Stechlin or Breiter Luzin were arbitrarily set to 1 km (i.e., log_10 _= 0). Stepwise Mantel tests were subsequently separately performed by first removing the two spring-spawning populations *C. lucinensis *and *C. fontanae *(8 lakes, *n *= 28 pairwise comparisons) and then removing the two *C. albula *populations from Lakes Stechlin and Breiter Luzin (6 lakes, *n *= 15 pairwise comparisons). These tests aimed to determine whether IBD was disrupted by inclusion of the sympatric populations.

### Differentiation and hybridization among sympatric pairs

We calculated pairwise *θ*^B ^using HICKORY in order to obtain 95% credibility intervals for differentiation among the four populations that occur as sympatric pairs (20,000 burn-in iterations, 100,000 final iterations). Furthermore, STRUCTURE 2.2 [40,55] was used to investigate possible gene flow between the sympatric populations within the two lakes, and to detect potential hybridization with stocked fish from Lakes Arend and Tollense. We assumed that most individuals of both populations each had pure ancestry but that a small proportion of individuals may have some ancestry from alternative populations. We set GENSBACK = 2 thus assuming that the mixed ancestry may have originated from the two previous generations. To ensure that there is strong statistical support for any inference of mixed ancestry, we set the prior probability of migrants to a low value (MIGRPRIOR = 0.01). The calculations were performed on the final 50,000 runs, after discarding the first 10,000 runs as a burn-in.

### Outlier loci

To evaluate those AFLP loci that may be subject to selection, a genome scan was conducted by assuming that genetic differentiation between sympatric populations is higher for those loci that are under divergent selection [4,72]. *F*_ST _and heterozygosity per polymorphic locus were calculated for the two population pairs and compared with simulated null distributions (at *p *= 0.95 and *p *= 0.99) using DFDIST[73,74]. The simulated quantiles and the locus-specific *F*_ST _were plotted against heterozygosity to determine those loci for which the neutral model could be rejected [73,74]. These outlier loci were compared between the two lakes. At *p *= 0.99, the probability that loci putatively under selection are shared in both lakes just by chance is 0.01 × 0.01 × 1264 = 0.1264. Accordingly, any locus with *p *> 0.995 or *p *< 0.005 found in both lakes would be a true outlier. At *p *= 0.95, up to three shared loci (0.05 × 0.05 × 1264 = 3.16) could be false positives.

Subsequently, we removed all loci putatively under positive selection in either Lakes Stechlin or Breiter Luzin at *p *= 0.95 (*n *= 95, *see *results), and again calculated pairwise *θ*^B ^using HICKORY in order to obtain 95% credibility intervals for differentiation among the four populations that occur as sympatric pairs (20,000 burn-in iterations, 100,000 final iterations). Furthermore, a NJ tree was calculated based on Nei's distance between all 10 populations, based on the reduced matrix of supposedly neutral AFLP loci.

## Authors' contributions

TM conceived of the study, carried out most of the statistical analyses and drafted the manuscript. KP carried out most of the molecular genetics studies. CE carried out statistical analyses. MTM carried out some statistical analyses and drafted the manuscript. BN established the molecular genetics protocol and carried out some molecular genetics studies. JF conceived of the study, and participated in its design and coordination and helped to draft the manuscript. All authors read and approved the final manuscript.

## Supplementary Material

Additional file 1 **Overview on pairwise *θ *between 10 populations of the *Coregonus albula *complex**. Matrix of pairwise *θ*, as calculated by ARLEQUIN. Allopatric populations consist exclusively of *C. albula*. Sympatric populations are named by lake origin and the lake-specific species names. Click here for file

Additional file 2 **Isolation-by-distance model of *Coregonus *populations**. Scatter plot between log_10 _direct geographical distances (km) and genetic distance (*θ*/1-*θ*) for the 10 populations of the *C. albula *complex in 8 lakes of north Germany (a), the eight *C. albula *populations in eight lakes at either allopatry or sympatry (b), and the six allopatric *C. albula *populations (c). Click here for file
