# Supplementary material for: Genetic population structure of sympatric and allopatric populations of Baltic ciscoes (Coregonus albula complex, Teleostei, Coregonidae)
Source: BMC Evol Biol. 2010 Mar 29;10:85. doi: 10.1186/1471-2148-10-85 (PMC2853541; doi:10.1186/1471-2148-10-85)
Supplement: Additional file 1 — Overview on pairwise θ between 10 populations of the Coregonus albula complex. Matrix of pairwise θ, as calculated by ARLEQUIN. Allopatric populations consist exclusively of C. albula. Sympatric populations are named by lake origin and the lake-specific species names. [file 1471-2148-10-85-S1.PDF]

Additional file 1: Overview on pairwise θ between 10 populations of the *Coregonus albula* complex, as calculated by ARLEQUIN.

Allopatric populations consist exclusively of *C. albula*. Sympatric populations are named by lake origin and the lake-specific species names.

	Brueckentin	Glambeck	Ploener	Tollense	Arend	Behler	Stechlin *C. fontanae*	Breiter Luzin *C. lucinensis*	Stechlin *C. albula*	Breiter Luzin *C. albula*
Brueckentin	0									
Glambeck	0.1341	0								
Ploener	0.2405	0.16758	0							
Tollense	0.04344	0.10002	0.20439	0						
Arend	0.40497	0.32148	0.24758	0.34909	0					
Behler	0.40554	0.30936	0.18813	0.33346	0.14381	0				
Stechlin *C. fontanae*	0.38315	0.28805	0.2595	0.33575	0.17963	0.22942	0			
Breiter Luzin *C. lucinensis*	0.37586	0.27928	0.26859	0.3255	0.17905	0.23461	0.0819	0		
Stechlin *C. albula*	0.29832	0.22733	0.23829	0.26202	0.17355	0.19936	0.08271	0.0858	0	
Breiter Luzin *C. albula*	0.2576	0.19954	0.22296	0.21901	0.18411	0.20886	0.10852	0.0612	0.04153	0
