# Supplementary figures and images for: Genetic population structure of sympatric and allopatric populations of Baltic ciscoes (Coregonus albula complex, Teleostei, Coregonidae)
Source: BMC Evol Biol. 2010 Mar 29;10:85. doi: 10.1186/1471-2148-10-85 (PMC2853541; doi:10.1186/1471-2148-10-85)

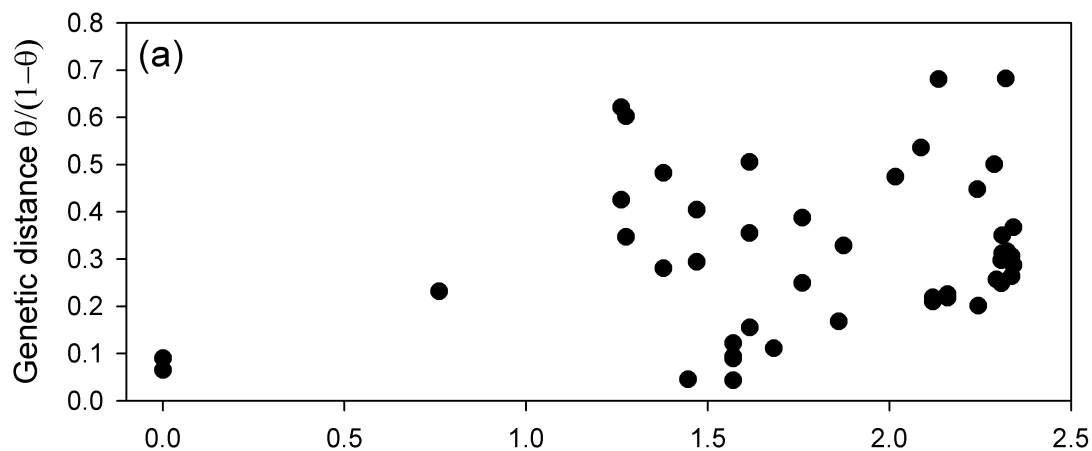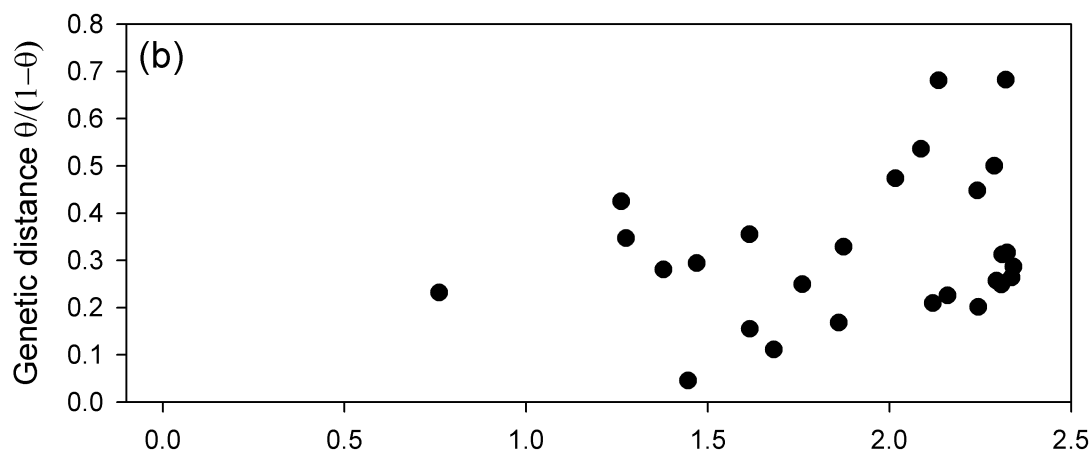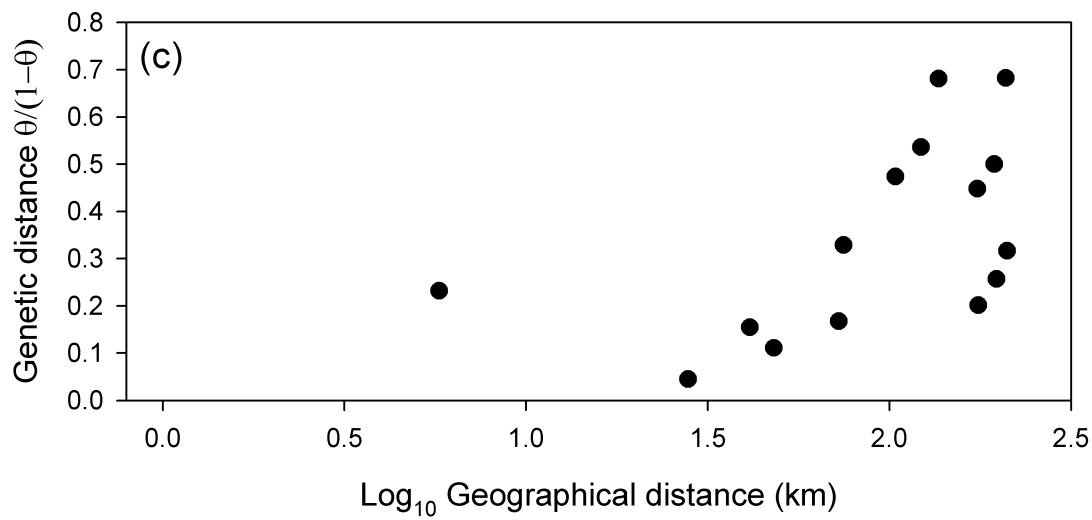

Supplement: Additional file 2 — Isolation-by-distance model of Coregonus populations. Scatter plot between log10 direct geographical distances (km) and genetic distance (θ/1-θ) for the 10 populations of the C. albula complex in 8 lakes of north Germany (a), the eight C. albula populations in eight lakes at either allopatry or sympatry (b), and the six allopatric C. albula populations (c). [file 1471-2148-10-85-S2.PDF]
